# Gadd45a deletion aggravates hematopoietic stem cell dysfunction in ATM-deficient mice

**DOI:** 10.1007/s13238-013-0017-9

**Published:** 2014-01-29

**Authors:** Yulin Chen, Runan Yang, Peng Guo, Zhenyu Ju

**Affiliations:** Institute of Aging Research, School of Medicine, Hangzhou Normal University, Hangzhou, 311121 China

**Keywords:** Gadd45a, ATM, hematopoietic stem cells, DNA damage

## Abstract

Ataxia telangiectasia mutated (ATM) kinase plays an essential role in the maintenance of genomic stability. ATM-deficient (ATM^−/−^) mice exhibit hematopoietic stem cell (HSC) dysfunction and a high incidence of lymphoma. Gadd45a controls cell cycle arrest, apoptosis and DNA repair, and is involved in the ATM-p53 mediated DNA damage response. However, the role of Gadd45a in regulating the functionality of ATM^−/−^ HSCs is unknown. Here we report that Gadd45a deletion did not rescue the defects of T-cells and B-cells development in ATM^−/−^ mice. Instead, ATM and Gadd45a double knockout (ATM^−/−^ Gadd45a^−/−^) HSCs exhibited an aggravated defect in long-term self-renewal capacity compared to ATM^−/−^ HSCs in HSC transplantation experiments. Further experiments revealed that the aggravated defect of ATM^−/−^ Gadd45a^−/−^ HSCs was due to a reduction of cell proliferation, associated with an accumulation of DNA damage and subsequent activation of DNA damage response including an up-regulation of p53-p21 signaling pathway. Additionally, ATM^−/−^ Gadd45a^−/−^ mice showed an increased incidence of hematopoietic malignancies, as well as an increased rate of metastasis than ATM^−/−^ mice. In conclusion, Gadd45a deletion aggravated the DNA damage accumulation, which subsequently resulted in a further impaired self-renewal capacity and an increased malignant transformation in ATM^−/−^ HSCs.

## Introduction

Hematopoietic stem cells (HSCs) are crucial to maintain the continuous regeneration of the blood and immune system throughout life (Orkin and Zon, 2008). Factors that affect HSC homeostasis may lead to blood and immune diseases. Previous studies have identified a significant role of ATM in the maintenance of HSCs and immune systems. ATM-deficient (ATM^−/−^) mice show progressive bone marrow failure and impaired HSC self-renewal due to an elevated level of reactive oxygen species (ROS) (Ito et al., 2004). Recent study showed that the ATM-BID pathway serves as a critical checkpoint in the homeostasis and DNA-damage-induced cell death of HSCs (Maryanovich et al., 2012). In addition to the stem cells in hematopoietic system, ATM also maintains the capacity of undifferentiated spermatogonia through suppressing the DNA damage accumulation and p21-mediated cell cycle arrest (Takubo et al., 2008).

Gadd45a is one of the DNA-damage checkpoint genes that have been implicated in ATM-p53 pathway (Zhan, 2005). Upon various kinds of stress, Gadd45a maintains genomic integrity in many cell types, through promoting cell death, cell cycle arrest, and DNA repair (Moskalev et al., 2012). Our recent study showed that Gadd45a is highly expressed in HSCs and regulates HSC stress responses. Gadd45a promotes DNA repair and eliminates damaged HSCs through apoptosis. However, the importance of Gadd45a in regulating HSC stress responses in the context of ATM deficiency is still unknown.

Here we employed the ATM and Gadd45a double knockout (ATM^−/−^ Gadd45a^−/−^) mice to investigate the role of Gadd45a in response to ATM deficiency in hematopoietic system. Our data showed that Gadd45a deletion did not rescue the hematopoietic defects in ATM^−/−^ mice. Instead, the ATM^−/−^ Gadd45a^−/−^ HSCs exhibited a significantly reduced reconstitution capacity compared to ATM^−/−^ HSCs in transplantation experiments. Further experiment revealed that Gadd45a deletion exacerbated the accumulation of DNA damage and the up-regulation of p53 and p21 expression in ATM^−/−^ HSCs. Interestingly, the ATM^−/−^ Gadd45a^−/−^ mice showed higher a incidence of lymphoma and leukemia, as well as an increased rate of metastasis compared to ATM^−/−^ mice.

## Results

### Gadd45a deletion does not rescue the defect of immune system in ATM^−/−^ mice

**Figure 1:**
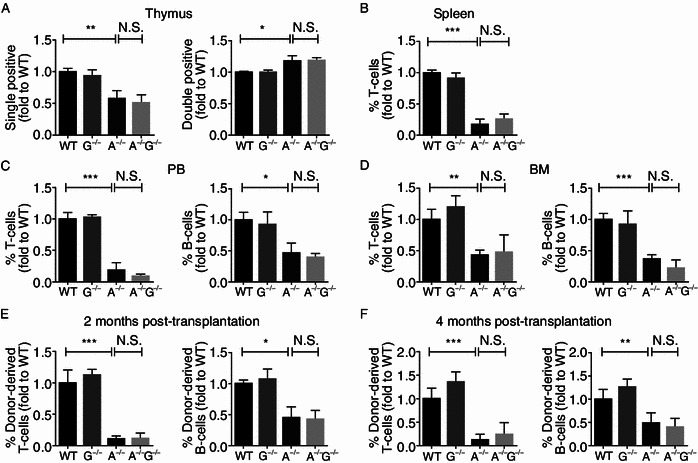
**Gadd45a deletion does not rescue the defect of immune system in ATM**^**−/−**^**mice**. (A–D) The WT, Gadd45a^−/−^, ATM^−/−^, and ATM^−/−^ Gadd45a^−/−^ mice (2–3 months old) were analyzed by FACS. The percentage of T-cells and/or B-cells (relative to WT) is shown in the indicated hematopoietic tissues. (*n* = 3–4). (E–F) The LSK cells (CD45.2) from WT, Gadd45a^−/−^, ATM^−/−^, and ATM^−/−^ Gadd45a^−/−^ mice were transplanted into lethally irradiated recipient mice (CD45.1). The percentage of donor-derived T-cells and B-cells in PB (relative to WT) was analyzed 2 and 4 months post-transplantation by FACS. (*n* = 3–4)

To investigate the role of Gadd45a in response to ATM deficiency, we crossed Gadd45a-deficient (Gadd45a^−/−^) mice with ATM^−/−^ mice to generate ATM^−/−^ Gadd45a^−/−^ mice. The hematopoiesis of wild-type (WT), Gadd45a^−/−^, ATM^−/−^, and ATM^−/−^ Gadd45a^−/−^ mice at age of 2–3 months was analyzed by FACS. Consistent with previous studies, the percentage of T-cells and/or B-cells in bone marrow (BM), peripheral blood (PB), spleen, and thymus of ATM^−/−^ mice was significantly lower than WT mice and Gadd45a^−/−^ mice (Fig. 1A–D). Deletion of Gadd45a did not rescue these defects (Fig. 1A–D).

To investigate whether the cell extrinsic factors might influence the phenotype of ATM^−/−^ Gadd45a^−/−^ mice, we transplanted HSCs from WT, Gadd45a^−/−^, ATM^−/−^, and ATM^−/−^ Gadd45a^−/−^ mice (donor, CD45.2) into lethally irradiated recipient mice (CD45.1) along with total bone marrow cells as competitor (CD45.1/2). Two and four months after transplantation, the percentage of donor-derived T-cells and B-cells in PB and BM of ATM^−/−^ and ATM^−/−^ Gadd45a^−/−^ mice was still comparable and much lower than WT and Gadd45a^−/−^ mice, which indicated that deletion of Gadd45a has no impact on the cell intrinsic defects of immune system of ATM^−/−^ mice (Fig. 1E and 1F).

### Gadd45a deletion aggravates the defect of self-renewal capacity of ATM^−/−^ HSCs

**Figure 2:**
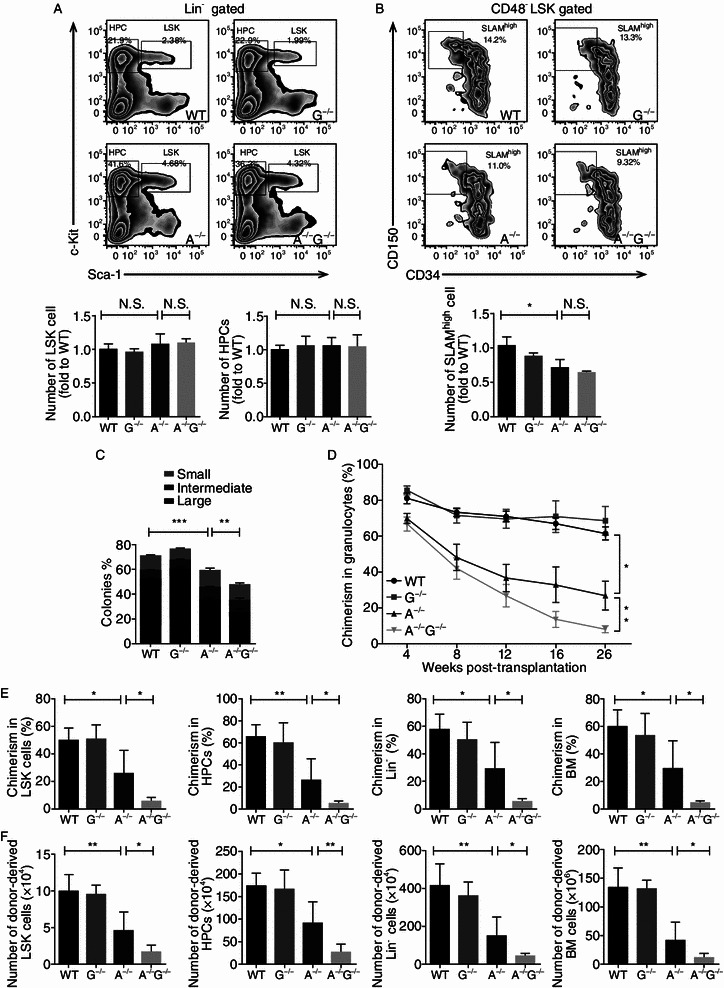
**Gadd45a deletion aggravates the defect of self-renewal capacity of ATM**^−**/**−^**HSCs**. (A–B) HSCs and HPCs from 2–3 months old mice were analyzed by FACS. The number of LSK cells, HPCs, and LT-HSCs (SLAM^high^) were analyzed and are shown (relative to WT). (*n* = 4). (C) HSCs were clonally sorted into 96-well plates (one cell per well) from WT, Gadd45a^−/−^, ATM^−/−^, and ATM^−/−^ Gadd45a^−/−^ mice and cultured in liquid medium for 14 days. The percentage of colonies was calculated by dividing the number of colonies with the original number of single-cell seeded. (*n* = 3). (D) Four thousand LSK cells (CD45.2) isolated from WT, Gadd45a^−/−^, ATM^−/−^, and ATM^−/−^ Gadd45a^−/−^ mice were transplanted into lethally irradiated recipients (CD45.1), along with competitors (CD45.1/2). The chimerism of donor-derived granulocytes in PB was tested monthly. The result is shown at indicated time point post-transplantation. (*n* = 5–8). (E) The chimerism of donor-derived indicated population in BM was tested 26 weeks post-transplantation. (*n* = 5–8). (F) The absolute number of the indicated donor-derived cells in BM was analyzed by FACS 26 weeks after transplantation. (*n* = 5–8)

Considering that Gadd45a mediates the stress-induced apoptosis and cell cycle arrest in many cell types, we investigate the impact of Gadd45a deletion in the maintenance and functionality of HSCs in ATM^−/−^ mice. The number of HSCs in WT, Gadd45a^−/−^, ATM^−/−^, and ATM^−/−^ Gadd45a^−/−^ mice at age of 2–3 months were analyzed by FACS. Consistent with previous report, the numbers of LSK cells (Lin^−^cKit^+^Sca1^+^, a population containing long-term HSCs, short-term HSCs, and multipotent progenitor cells) and hematopoietic progenitor cells (HPCs, Lin^−^cKit^+^Sca1^−^) is comparable in ATM^−/−^ mice and WT mice at young age (Fig. 2A). Interestingly, we observed a significant reduction in the numbers of long-term HSCs (SLAM^high^ HSCs, i.e. CD34^−^CD48^−^CD150^high^LSK) in ATM^−/−^ mice compared to WT mice (Fig. 2B). However, Gadd45a deletion did not influence the maintenance of HSCs and HPCs in both WT and ATM^−/−^ mice (Fig. 2A and 2B). These data indicated that Gadd45a deletion does not improve the HSC maintenance in ATM^−/−^ mice.

To investigate whether Gadd45a deletion has a functional impact on the colony-forming capacity of ATM^−/−^ HSCs, we conducted single-cell colony forming assay. HSCs were clonally sorted into 96-well plates from WT, Gadd45a^−/−^, ATM^−/−^, and ATM^−/−^ Gadd45a^−/−^ mice and cultured in liquid medium supplied with cytokines for 14 days. As expected, the colony forming capacity of ATM^−/−^ HSCs was significantly decreased as compared to WT (Fig. 2C). Interestingly, the ATM^−/−^ Gadd45a^−/−^ HSCs formed even fewer colonies than ATM^−/−^ HSCs, suggesting Gadd45a deletion impairs the colony forming capacity of ATM^−/−^ HSCs (Fig. 2C).

To evaluate whether Gadd45a deletion has impact on the long-term reconstitution ability of ATM^−/−^ HSCs, we conducted competitive HSC transplantation assay. Four thousand LSK cells from WT, Gadd45a^−/−^, ATM^−/−^, and ATM^−/−^ Gadd45a^−/−^ mice (donor, CD45.2) were transplanted into lethally irradiated recipient mice (CD45.1) along with total bone marrow cells (competitor, CD45.1/2). The chimerism of donor-derived granulocytes in PB, as an indicator of HSC reconstitution ability, was analyzed at indicated time point post-transplantation (Fig. 2D). As expected, the PB chimerism of ATM^−/−^ donor-derived granulocytes was significantly lower than WT and Gadd45a^−/−^ controls. The PB chimerism of ATM^−/−^ Gadd45a^−/−^ donor-derived granulocytes was comparable to the ATM^−/−^ group 4–12 weeks post-transplantation. However, the PB chimerism of ATM^−/−^ Gadd45a^−/−^ donor-derived granulocytes showed a progressive reduction than the ATM^−/−^ group 16–26 weeks post-transplantation. This data indicate that Gadd45a deletion does not influence the short-term reconstitution capacity of ATM^−/−^ HSCs and HPCs but significantly aggravates the long-term reconstitution defect of ATM^−/−^ HSCs (Fig. 2D).

To figure out whether the aggravated defect in the long-term reconstitution ability of ATM^−/−^ Gadd45a^−/−^ HSCs resulted from an impaired self-renewal capacity, we analyzed the donor-derived HSCs in the BM of recipient mice 26 weeks after transplantation. The chimerism of ATM^−/−^ donor-derived cells was significantly lower than the chimerism of WT and Gadd45a^−/−^ donor-derived cells in the compartment of total BM cell, Lineage negative BM cells, HPCs, and HSCs in the recipient mice (Fig. 2E). ATM^−/−^ Gadd45a^−/−^ donor-derived cells showed a further reduction of the chimerism in the compartment of total BM cell, Lineage negative BM cells, HPCs, and HSCs compared to ATM^−/−^ donor-derived cells, suggesting that Gadd45a deletion aggravates the competitive regenerative capacity of ATM^−/−^ HSCs (Fig. 2E). Since the same amount of donor HSC has been transplanted to the recipient mice, the absolute numbers of donor-derived HSCs 26 weeks after transplantation can be used to assess the actual self-renewal of the HSCs. The number of ATM^−/−^ donor-derived HSCs, HPCs, Lineage^−^ cells, and total BM cells was all significantly reduced compared to their counterparts in the WT and Gadd45a^−/−^ group (Fig. 2F). Additionally, the ATM^−/−^ Gadd45a^−/−^ donor-derived HSCs, HPCs, Lineage^−^ cells, and total BM cells were all significantly reduced compared to their counterparts in the ATM^−/−^ group (Fig. 2F). Taken together, these data indicated that Gadd45a deletion impairs the self-renewal capacity of ATM^−/−^ HSCs, which result in the aggravated defect in the reconstitution capacity of ATM^−/−^ Gadd45a^−/−^ HSCs.

### Reduced proliferation in ATM^−/−^ Gadd45a^−/−^ HSCs after transplantation

**Figure 3:**
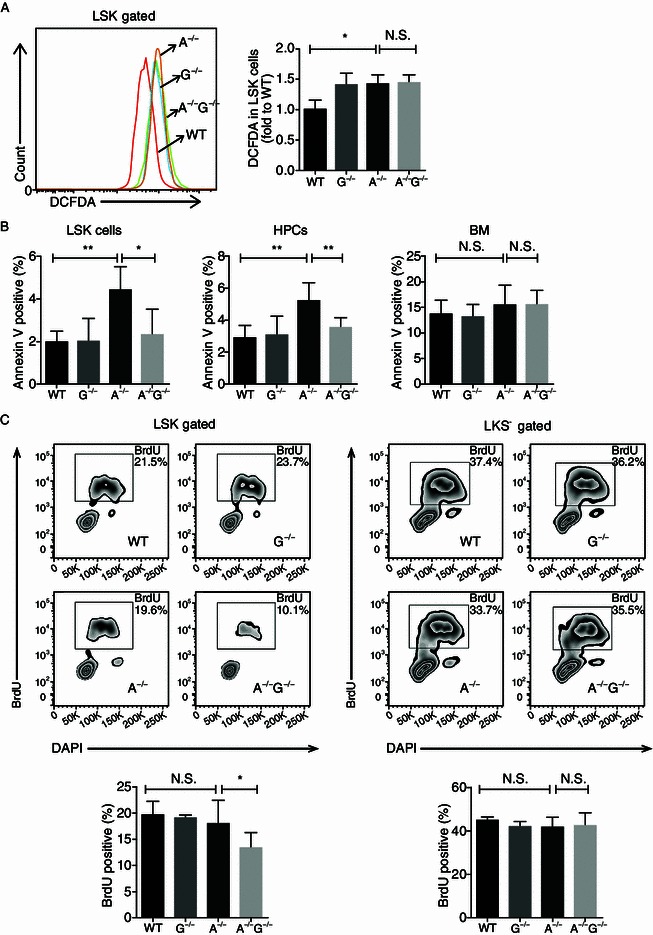
**Reduced proliferation in ATM**^−**/**−^**Gadd45a**^−**/**−^**HSCs after transplantation**. (A–C) ROS level, apoptosis, and cell cycle of donor-derived cells were detected 26 weeks after transplantation. (A) ROS level was detected by the fluorescence intensity of DCFDA in LSK cells. The representative FACS plots are shown. (*n* = 3). (B) Apoptosis of the indicated population was tested by Annexin V/DAPI staining. The percentage of Annexin V positive population was compared in each group. (*n* = 5–7). (C) Cell cycle of HSCs (LSK gated) and HPCs (LKS^−^ gated) was examined by BrdU incorporation. The percentage of BrdU positive population was compared in each group. (*n* = 5–7)

It has been reported that ATM functions as a sensor of ROS (Guo et al., 2010), therefore ATM deficiency results in an elevated level of ROS, which is responsible for the impaired self-renewal of ATM^−/−^ HSCs (Ito et al., 2004). To investigate the underlying mechanism of the aggravated self-renewal defect of ATM^−/−^ Gadd45a^−/−^ HSCs, we examined the ROS level in HSCs by staining of 2'-7'-dichlorofluorescene diacetate (DCFDA). As expected the ROS level of ATM^−/−^ HSCs was higher than WT HSCs. However, ATM^−/−^ Gadd45a^−/−^ HSCs and ATM^−/−^ HSCs showed a comparable ROS level, suggesting that the ROS level is not responsible for the aggravated self-renewal capacity of ATM^−/−^ Gadd45a^−/−^ HSCs (Fig. 3A). In addition, we found an elevated level of ROS in Gadd45a^−/−^ HSCs, implicating that Gadd45a alone might have a functional role in regulating the ROS level in HSCs.

Since ATM has an important role in DNA damage checkpoint responses (Lavin et al., 2005), we then examined the apoptosis and proliferation in HSCs, HPCs, and BM cells from WT, Gadd45a^−/−^, ATM^−/−^, and ATM^−/−^ Gadd45a^−/−^ mice 26 weeks after transplantation. Apoptosis was determined by Annexin V/DAPI staining. The percentage of apoptotic cells in ATM^−/−^ Gadd45a^−/−^ HSCs and HPCs was significantly lower than the ATM^−/−^ counterparts, indicating that Gadd45a deletion improves the survival of ATM^−/−^ HSCs and HPCs (Fig. 3B). Next, we examined the cell proliferation by BrdU incorporation assay. The percentage of BrdU positive cells was remarkably reduced in ATM^−/−^ Gadd45a^−/−^ HSCs than ATM^−/−^ HSCs, whereas the proliferation was comparable in ATM^−/−^ Gadd45a^−/−^ HPCs and ATM^−/−^ HPCs (Fig. 3C). These data indicate that Gadd45a deletion impairs the proliferation of ATM^−/−^ HSCs, which is responsible for the aggravated defect of HSC self-renewal in ATM^−/−^ Gadd45a^−/−^ mice.

### Increased DNA damage and p53-p21 activation in ATM^−/−^ Gadd45a^−/−^ HSCs after transplantation

**Figure 4:**
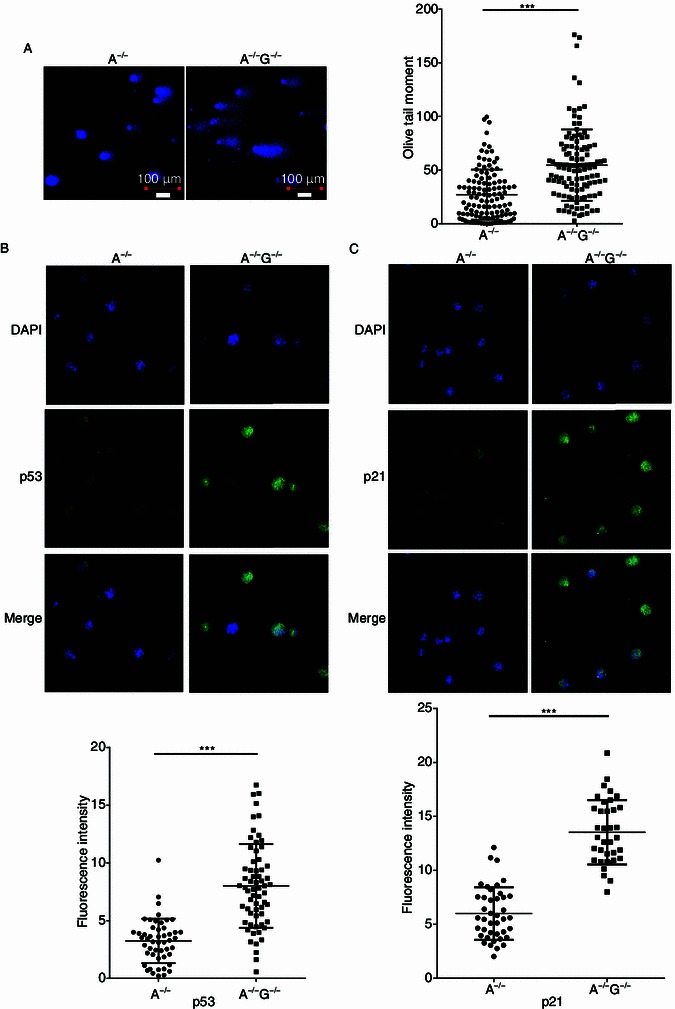
**DNA-damage-induced p53-p21 activation in ATM**^−**/**−^**Gadd45a**^−**/**−^**HSCs after transplantation**. (A–C) ATM^−/−^ and ATM^−/−^ Gadd45a^−/−^ donor-derived LSK cells were isolated by FACS sorting 26 weeks after transplantation and subjected to fluorescence experiments. (A) The DNA damage accumulation was assessed by comet assay. The representative photos and statistical results are shown. The Olive Tail Moment stands for DNA damage level per cell. (*n* = 117). (B–C) The expression of p53 and p21 in ATM^−/−^ and ATM^−/−^ Gadd45a^−/−^ donor-derived LSK cells in single-cell level was compared by immunofluorescence. The fluorescence intensity stands for expression level in single cell, which was determined by ImageJ (designed by NIH). (*n* = 40–60)

To further investigate the potential molecular mechanism of the reduced proliferative capacity of ATM^−/−^ Gadd45a^−/−^ HSCs after transplantation, we compared the DNA damage level of donor-derived ATM^−/−^ HSCs and ATM^−/−^ Gadd45a^−/−^ HSCs 26 weeks after transplantation through comet assay. The data showed a significant increase of DNA damage in ATM^−/−^ Gadd45a^−/−^ HSCs compared to ATM^−/−^ HSCs, indicating Gadd45a deletion leads to a further impairment in DNA repair in ATM^−/−^ HSCs (Fig. 4A). Next, we examined the expression level of p53 and p21 in FACS purified ATM^−/−^ and ATM^−/−^ Gadd45a^−/−^ HSCs by immunofluorescence staining. The ATM^−/−^ Gadd45a^−/−^ HSCs showed a significantly increase of p53 and p21 fluorescence intensity compared to ATM^−/−^ HSCs (Fig. 4B and 4C). These data suggest that Gadd45a deletion results in an increased level of DNA damage in ATM^−/−^ HSCs, which then activates p53-p21-mediated cell cycle arrest, and subsequently inhibit the self-renewal capacity of ATM^−/−^ Gadd45a^−/−^ HSCs.

### Gadd45a prevents the incidence of hematopoietic malignancies in ATM^−/−^ mice

**Figure 5:**
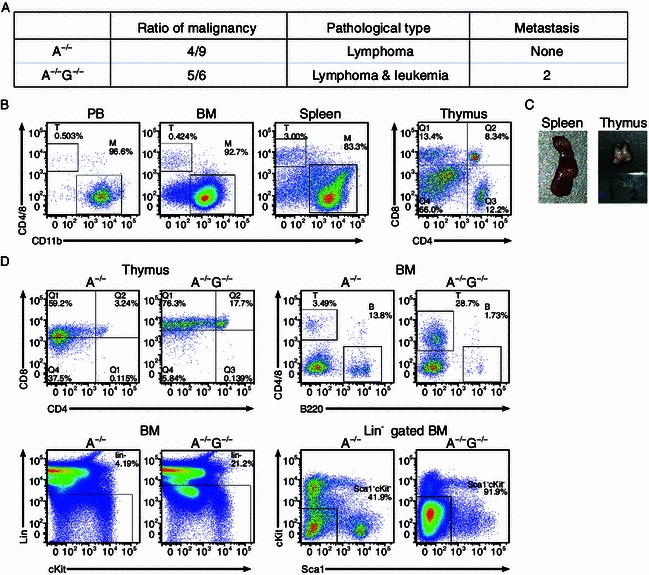
**Gadd45a prevents the incidence of hematopoietic malignancies in ATM**^−**/**−^**mice**. (A) The ratio of malignancy, pathological type, and metastasis in ATM^−/−^ and ATM^−/−^ Gadd45a^−/−^ mice are shown. (B–C) AML with spleen infiltration was observed in one ATM^−/−^ Gadd45a^−/−^ mice (without lymphoma). The FACS plots in the indicated tissues and the pictures of spleen (infiltrated) and thymus (without lymphoma) of the AML mice are shown. (D) Representative FACS plots in the indicated tissues in ATM^−/−^ (lymphoma without BM infiltration) and ATM^−/−^ Gadd45a^−/−^ (lymphoma with BM infiltration) mice with are shown

Previous study showed a high incidence of lymphoma in ATM^−/−^ mice (Xu et al., 1996). In our study, ATM^−/−^ mice only developed T-cell lymphoma. Interestingly, ATM^−/−^ Gadd45a^−/−^ mice not only showed a higher incidence of T-cell lymphoma compared to ATM^−/−^ mice (Fig. 5A), but also developed acute myeloid leukemia (AML) (Fig. 5B and 5C). Furthermore, we also observed an increased metastasis (one case of BM infiltration and one case of spleen infiltration) in ATM^−/−^ Gadd45a^−/−^ mice, which indicates that Gadd45a deletion increases the degree of malignancy of lymphoma in ATM^−/−^ mice (Fig. 5D). These data suggested that Gadd45a deletion not only increases the susceptibility of malignant transformation but also expands the spectrum of hematopoietic malignancy in ATM^−/−^ Gadd45a^−/−^ mice.

## Discussion

ATM is known as a sensor of DNA damage and oxidative stress to initiate various responses through phosphorylation of many checkpoint proteins, including γ-H2AX, p53, and CHK2 (Banin et al., 1998; Friesner et al., 2005; Kastan et al., 1992; Matsuoka et al., 2000). ATM deficiency leads to severe defects in many tissues and a higher incidence of lymphoma in mice probably through an elevated ROS level and an increased genomic instability (Shiloh and Kastan, 2001). As a target of ATM-p53 signaling pathway, Gadd45a plays a very important role in regulating the responses to various types of stress (Kastan et al., 1992; Zhan, 2005). However, it is still unknown whether Gadd45a mediates some of the deleterious effects of ATM deficiency. In this study, we found that Gadd45a deletion did not rescue the hematopoietic defects in ATM^−/−^ mice, but rather aggravated the compromised self-renewal capacity of HSCs and the malignant transformation in ATM^−/−^ mice.

It has been previously reported that the increased level of ROS and subsequent activation of p38 and de-repression of p16 are responsible for the impaired self-renewal of ATM^−/−^ HSCs (Ito et al., 2004; Ito et al., 2006). In our study, the deletion of Gadd45a led to an increased level of ROS in ATM^+/+^ HSCs but not in ATM^−/−^ HSCs, suggesting that an ATM-dependent function of Gadd45a in regulating oxidative stress in HSCs. Therefore, the aggravated HSC self-renewal capacity in response to Gadd45a deletion is not due to its role in regulating oxidative stress.

In addition to the role of regulating oxidative stress, ATM has also been implicated in the maintenance of genomic stability in stem cells. In spermatogonia, ATM deficiency induces cell cycle arrest through activation of p19-p53-p21 pathway, leading to the defect of stem cell function (Takubo et al., 2008). Indeed, our data showed that Gadd45a deletion resulted in a significant increase in the activation of p53-p21 signaling pathway in the ATM^−/−^ HSCs, due to an increased level of DNA damage. Therefore, our study suggested that Gadd45a has a unique role in preventing the exhaustion of the self-renewal capacity in ATM deficient HSCs, probably through promoting DNA repair and maintaining genomic integrity in ATM^−/−^ HSCs.

Aberrant DNA damage responses and increased accumulation of DNA damage has been implicated in malignant transformation in the hematopoietic system. In line with this, our data showed that deletion of Gadd45a increased the incidence of T-cell lymphoma in ATM^−/−^ mice. In addition, the incidence of metastasis was also increased in ATM^−/−^ Gadd45a^−/−^ mice, indicating a role of Gadd45a in the prognosis of hematopoietic malignancies. More interestingly, we also observed acute myeloid leukemia (AML) in ATM^−/−^ Gadd45a^−/−^ mice. Myeloid leukemia has been extremely rare in both ATM deficient mice and AT patients (Onoda et al., 2013). ATM^−/−^ mice mainly developed thymic lymphoma through an aberrant T cell receptor alpha/delta rearrangement and gene amplification (Zha et al., 2010), whereas AML is a clonal disease originated from stem cell and/or progenitor cell that often associated with genomic aberrations and chromosomal rearrangement. Therefore, our data suggested that Gadd45a is important in the maintenance of DNA repair and genomic integrity in ATM^−/−^ HSCs and prevents malignant transformation at stem cell level.

In conclusion, Gadd45a serves as an important regulator in maintaining HSC self-renewal capacity and mitigating hematopoietic malignancy in the context of ATM deficiency. These findings underscore the importance of the DNA damage checkpoint genes in the regulation of HSC function and the prevention of malignant transformation through the maintenance of genomic integrity at stem cell level. Our study provides important experimental evidence for the clinical applications in the future.

## Materials and Methods

### Mice

Gadd45a^−/−^ mice were a kind gift from Professor Albert J. Fornace, Jr. and ATM^−/−^ mice were purchased from the Jackson Laboratory. Both were maintained in a C57BL/6 (CD45.2) background. The recipient mice used in the competitive transplantation assays were either CD45.1 mice or CD45.1/CD45.2 mice. The Animal Care and Ethics Committee at Hangzhou Normal University approved all animal experiments in our study.

### Flow cytometry

Antibodies used for flow cytometry analysis or cell sorting were purchased from eBioscience, Biolegend or BD Biosciences. For HSC staining, the BM cells are incubated with the lineage antibody mixture including CD11b, Gr1, B220, CD4, CD8, Ter119 for 30 min, and then stained by other surface makers. After staining, red blood cells were eliminated by lysing buffer (BD Biosciences) and analyzed on a flow cytometer (LSRFortessa, BD Biosciences). For cell sorting, total BM cells were stained with an anti-mouse c-Kit, followed by enrichment with anti-APC micro-beads (Miltenyi Biotec); the enriched cells were then stained with surface markers and sorted by flow cytometry (Influx, BD Biosciences) for further *in vivo* and *in vitro* investigations.

### Single-cell colony forming assay

Single cells were cultured for 14 days in liquid medium supplemented with 10% fetal bovine serum (FBS, Life technologies), 20% BIT 9500 (Stemcell Technologies), 2 mmol/L L-glutamine (Life technologies), 100 U/mL penicillin/streptomycin, 5 × 10^−5^ mol/L β-ME (Sigma-Aldrich), 10 ng/mL stem cell factor (SCF, Pepro Tech), 10 ng/mL thrombopoietin (TPO, Pepro Tech), and 10 ng/mL interleukin-3 (IL-3, Pepro Tech). Three classes of colonies were defined: large colonies consisting of more than 10,000 cells, intermediate colonies consisting of more than 1000 cells, small colonies consisting of more than 100 cells.

### Competitive transplantation assay

Four thousand LSK cells freshly isolated from WT, Gadd45a^−/−^, ATM^−/−^, and ATM^−/−^ Gadd45a^−/−^ mice (donor, CD45.2) are transplanted into lethally irradiated recipient mice (CD45.1) along with total bone marrow cells (competitor, CD45.1/2). The chimerism of donor-derived granulocytes in PB, as an indicator of HSC reconstitution ability, is analyzed at the indicated time point post-transplantation.

### Cell cycle analysis

For BrdU incorporation, mice were injected with BrdU by i.p. 1–2 h before euthanasia. After surface-marker staining, BM cells were fixed and stained with the anti-BrdU antibody, along with DAPI counter staining, using the BrdU Flow Kit (BD Pharmingen). Prepared samples were then analyzed by FACS on LSRFortessa (BD Biosciences).

### Apoptosis

For apoptosis analysis, after surface-marker staining, BM cells were washed and stained with Annexin V/DAPI, using an Annexin V Apoptosis Detection Kit (eBioscience or BD Pharmingen). Prepared samples were then analyzed by FACS on LSRFortessa (BD Biosciences).

### Immunofluorescence

Sorted cells were placed on poly-L-lysine coated slides, fixed with 4% paraformaldehyde, permeabilized by 0.3% Triton-X 100, and blocked by 2% BSA-PBS. p53 or p21 antibody (Santa Cruz Biotechnology) was used at 1:200 in the blocking solution. The secondary antibody was incubated for 30 min (Life Technologies). Lastly, DAPI was used for nuclear staining. The slides were then mounted in a mounting medium (Vector Laboratories) and visualized and photographed using a confocal fluorescence microscope (LSM 710, Carl Zeiss International).

### Comet assay

The alkaline comet assay was performed to evaluate the degree of DNA damage. Briefly, cells were mixed with 1% low-temperature-gelling agarose (Sigma-Aldrich) in a 1:3 ratio and spread on slides pre-coated with 1% agarose gel (Life Technologies). The prepared samples were lysed for 1 h and rinsed in DNA-unwinding solution for 20–40 min. The samples were then subjected to electrophoresis, stained with DAPI and observed and photographed using a confocal fluorescence microscope.

### Statistical analysis

Data are presented as mean ± SD. The statistical significance of the differences between groups was calculated using the unpaired Student’s *t*-test, and is displayed as *P* < 0.05 (one asterisk), *P* < 0.01 (two asterisks), or *P* < 0.001 (three asterisks).

## Abbreviations

AML: acute myeloid leukemia
ATM: Ataxia telangiectasia mutated
BM: bone marrow
HPC: hematopoietic progenitor cell
HSC: hematopoietic stem cell
PB: peripheral blood
ROS: reactive oxygen species
WT: wild-type
